# Time‐Conditioned Zero‐Shot Self‐Supervised Reconstruction for Accelerated 3D Ultra‐Low‐Field MRI

**DOI:** 10.1002/mrm.70407

**Published:** 2026-04-28

**Authors:** Mart W. J. van Straten, Beatrice Lena, Chloé Najac, Ruben van den Broek, Peter Börnert, Andrew Webb, Yiming Dong

**Affiliations:** ^1^ C.J. Gorter MRI Center, Department of Radiology LUMC Leiden the Netherlands; ^2^ Department of Biomedical Engineering Eindhoven University of Technology Eindhoven the Netherlands; ^3^ Philips Innovative Technologies Hamburg Hamburg Germany

**Keywords:** 3D image reconstruction, physics‐guided deep learning, ultra‐low‐field MRI, undersampled MRI, zero‐shot self‐supervised learning

## Abstract

**Purpose:**

Ultra‐low‐field (ULF) MRI provides a cost‐effective, portable imaging option but has relatively low SNR and long acquisition times compared to standard clinical scans. This study presents a time‐conditioned zero‐shot self‐supervised learning image reconstruction framework (ULF‐ZS‐SSL) to accelerate 3D‐acquired single‐coil ULF MRI without relying on external training data. In addition, for faster computation, a transfer‐learning (TL) variant (ULF‐ZS‐SSL‐TL) was implemented by pretraining on a small fully‐sampled ULF brain dataset and fine‐tuning on the target subject in a zero‐shot manner.

**Methods:**

This image reconstruction method combines a physics‐based data‐consistency step with a 3D residual network prior and sinusoidal time‐step embeddings to improve convergence speed. Data were acquired on a 47 mT Halbach‐based scanner using 3D turbo spin‐echo sequences with T_1_‐, T_2_‐, and inversion‐recovery–T_1_‐weighted contrasts. Additional T_1_‐weighted wrist scans were acquired to evaluate cross‐anatomy generalization. Both true and retrospectively undersampled data were compared with total variation (TV) and model‐based deep learning (MoDL).

**Results:**

The ULF‐ZS‐SSL method produced high‐quality reconstructions across all tested contrasts, outperforming zero‐filled and TV reconstructions, particularly at higher acceleration factors. Time‐step conditioning improved convergence speed, while ULF‐ZS‐SSL‐TL further accelerated the image reconstruction three‐fold, enabling full 3D reconstructions in about 3 min. Pretraining on brain data also worked well for wrist reconstructions, indicating cross‐anatomy generalization.

**Conclusion:**

The ULF‐ZS‐SSL framework enables accurate, training‐free reconstruction of undersampled single‐coil ULF MRI data, as does the ULF‐ZS‐SSL‐TL approach using minimal training data. The combination of physics‐based unrolling, time‐step conditioning, and transfer‐learning supports rapid and robust application in portable or resource‐limited ULF MRI systems.

## 1 Introduction

Portable low‐field MRI, typically operating below 0.2 T, has recently emerged as a promising cost‐effective imaging modality for global health applications [1, 2, 3, 4, 5]. Such systems, based on compact permanent magnets, can be deployed in diverse environments ranging from community clinics and rural hospitals to ambulances and emergency settings, where traditional scanners are impractical or entirely unavailable. However, these advantages come at the cost of substantially reduced SNR compared to conventional high‐field MRI, resulting in long acquisition times and reduced spatial resolution. Accelerated acquisitions followed by advanced reconstruction methods are therefore essential for ULF MRI [6].

Undersampling of k‐space is a key strategy in MRI and is widely used at high‐field to reduce scan time [7, 8, 9, 10]. However, reconstructing images from undersampled data remains particularly challenging at low‐field when only a single transmit/receive coil element is typically used [3, 11, 12], eliminating the possibility of leveraging parallel imaging techniques [8, 9].

Deep learning‐based image reconstruction has demonstrated remarkable performance in accelerating MRI across a range of field strengths [13]. However, the quality of supervised learning‐based reconstruction [14, 15], in particular, is critically dependent on the availability of large, high‐quality training datasets that match the acquisition characteristics of the target domain. For ULF MRI, this requirement is difficult to meet. Data distributions differ not only with scanner hardware and field strength but also across highly variable acquisition environments [1, 2, 3, 4, 5, 11]. Moreover, intrinsic differences in tissue relaxation times (T_1_, T_2_) across organs further complicate direct model transfer from networks trained at higher fields (e.g., 1.5 or 3 T). It is difficult to bridge this gap through data simulation or artificial downsampling [16, 17, 18], which makes fully supervised learning particularly challenging for ULF MRI reconstruction.

To address this data scarcity, recent work introduced self‐supervision via data undersampling (SSDU [19]), a learning strategy that removes the dependency on fully‐sampled data. Among these, the zero‐shot self‐supervised learning (ZS‐SSL [20]) framework formulates reconstruction as a self‐consistency optimization performed directly on the undersampled dataset. The network learns an implicit prior by partitioning k‐space into disjoint subsets used for training, loss computation, and validation, thereby adapting uniquely to each subject and acquisition.

In this work, we implement the ZS‐SSL framework [20] as a time‐conditioned unrolled reconstruction tailored for 3D‐acquired ULF MRI (ULF‐ZS‐SSL). The proposed model integrates physics‐based data‐consistency (DC) with a 3D residual convolutional network (ResNet) acting as a learned prior, enabling recovery of high‐fidelity images from undersampled k‐space data. To further improve the efficiency of the original zero‐shot approach, we introduce a time‐embedding [21, 22, 23] mechanism to facilitate faster convergence. Additionally, the effect of transfer‐learning (TL) is investigated, in which the network is first pretrained on a small set of fully‐sampled low‐field data and subsequently fine‐tuned in a zero‐shot manner on the target subject (ULF‐ZS‐SSL‐TL). This approach preserves the self‐adaptive capability of zero‐shot optimization while substantially accelerating convergence. Through retrospective and prospective experiments on a 47 mT Halbach‐based scanner, we demonstrate that the proposed method reconstructs 3D turbo spin‐echo (TSE) based inversion‐recovery–T_1_‐weighted (IR‐T_1_w), T_1_‐weighted (T_1_w), and T_2_‐weighted (T_2_w) brain images with improved detail preservation and reduced artifacts compared with conventional total variation (TV [10]) and supervised model‐based deep learning (MoDL [14]) reconstructions. Furthermore, we validate the proposed method on T_1_‐weighted images of the wrist acquired on the same system to illustrate its potential applicability across different scan conditions and anatomies.

## 2 Theory

### 2.1 Physics‐Informed Supervised Learning for ULF MRI Reconstruction

In conventional supervised learning approaches, the objective is to learn a mapping between undersampled single‐coil k‐space data y and the fully‐sampled image x using a large dataset of paired examples. The forward model can be written as:

(1) y=MFx+n,

where ***n*** represents measurement noise, M denotes the sampling mask operator, and F is the Fourier transform operator. In physics‐informed deep learning‐based MRI reconstruction [14], the imaging inverse problem is addressed using an unrolled supervised network G(·;θ) with trainable parameters θ. For each unrolled iteration k, the reconstruction alternates between a learned regularization block g(·;θ) to obtain the intermediate result $x_{k+1/2}$ and a DC update:

(2) $$x_{k+1/2}=g\left(x_{k};\theta \right),$$

(3) $$x_{k+1}=\arg\min_{x}\;{\left\|\mathrm{MF}x-y\right\|}_{2}^{2}+\lambda {\left\|x-x_{k+1/2}\right\|}_{2}^{2},$$

where λ controls the strength of the constraint. The network parameters are trained by minimizing a loss function L(·,·) over many training pairs $\left(y_{i},x_{i}\right)$:

(4) $$\min_{\theta }\;\sum_{i}\;L\left(G\left(y_{i};\theta \right),x_{i}\right),$$

where i indexes individual subjects and $G\left(y_{i};\theta \right)$ denotes the output reconstruction of the unrolled network. Supervised training requires large domain‐matched datasets, which are difficult to obtain for ULF MRI.

### 2.2 Self‐Supervised Learning (SSL)

The SSDU strategy as proposed by Yaman et al. [19] removes the need for reference images $x_{i}$ by splitting the acquired k‐space data (Ω) into two complementary subsets for training ($\Omega_{t}$) and loss calculation ($\Omega_{l}$). The network is trained to reconstruct the k‐space data in $\Omega_{l}$ from those in $\Omega_{t}$ with the following objective function:

(5) $$\min_{\theta }\;\sum_{i}\;L\left(M_{\Omega_{l,i}}\mathrm{FG}\left(M_{\Omega_{t,i}}y_{i};\theta \right),M_{\Omega_{l,i}}y_{i}\right),$$

where $M_{\Omega_{t,i}}$ and $M_{\Omega_{l,i}}$ are binary sampling operators associated with the training and loss masks, respectively. Although no ground‐truth is required, SSDU still requires pretraining over many subjects to learn generalized priors.

### 2.3 Zero‐Shot Self‐Supervised Learning (ZS‐SSL)

**FIGURE 1:**
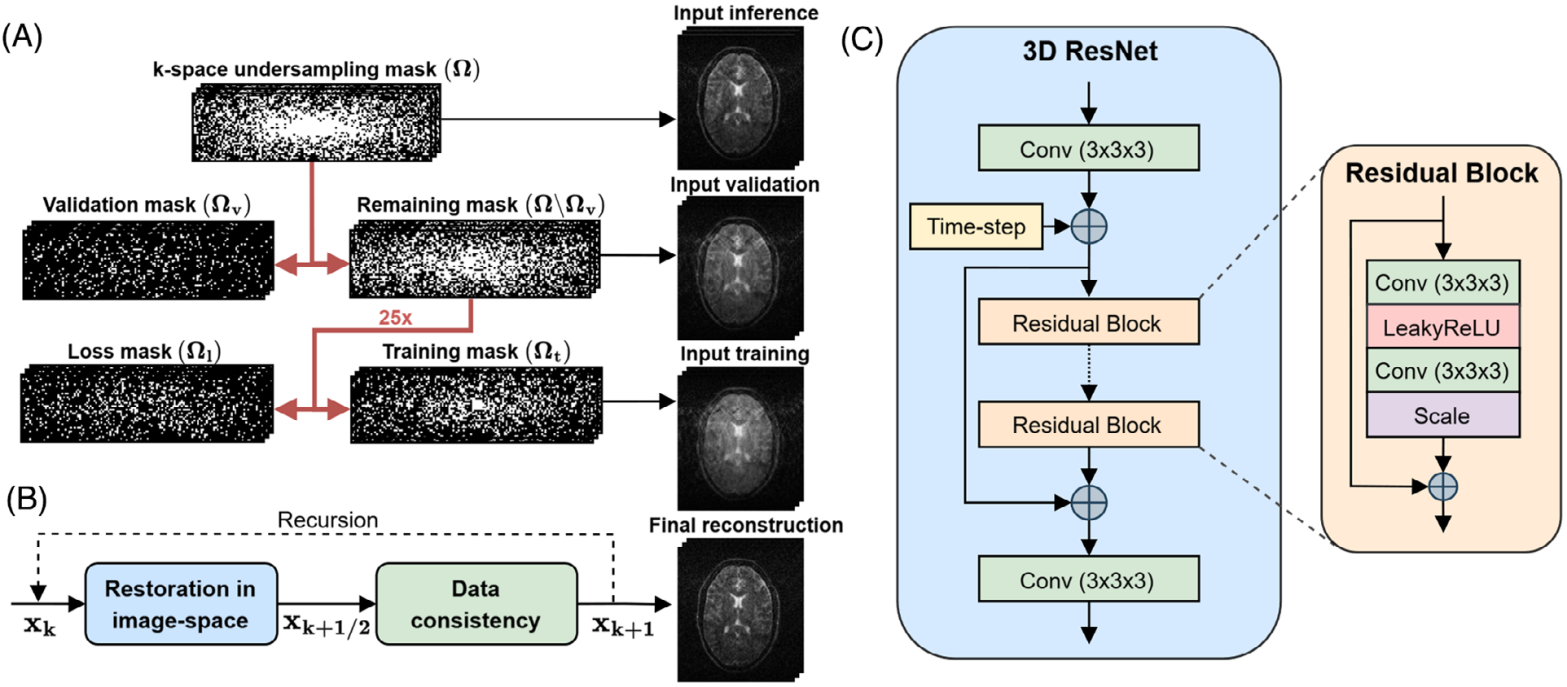
Overview of the ultra‐low‐field zero‐shot self‐supervised learning reconstruction framework (ULF‐ZS‐SSL) [20]. (a) The acquired undersampled k‐space (Ω) is randomly partitioned into three non‐overlapping subsets: a training mask ($\Omega_{t}$) used for data‐consistency (DC) enforcement, a loss mask ($\Omega_{l}$) used to compute the self‐supervised loss, and a validation mask ($\Omega_{v}$) used for early stopping. The training and loss partitioning is randomly re‐sampled 25 times to improve generalization. (b) Reconstruction is performed through an unrolled optimization that alternates between image‐domain restoration and physics‐based DC solved by conjugate‐gradient in 10 iterations. (c) The restoration module is implemented as a time‐conditioned 3D ResNet consisting of residual blocks with 3 × 3 × 3 convolutions, LeakyReLU activation, and a scaling factor (0.1). The sinusoidal time‐step embedding encodes the iteration index to guide progressive reconstruction across unrolled steps.

The ZS‐SSL formulation addresses this by learning entirely from the target dataset, eliminating dependence on external data. Following the formulation in Yaman et al. [20], the acquired k‐space data (Ω) is partitioned into training ($\Omega_{t}$), loss ($\Omega_{l}$), and validation ($\Omega_{v}$) subsets (Figure 1a). The training and loss subsets are randomly re‐sampled N times to improve generalization. The objective function becomes:

(6) $$\min_{\theta }\;\sum_{n=1}^{N}\;L\left(M_{\Omega_{l,n}}\mathrm{FG}\left(M_{\Omega_{t,n}}y;\theta \right),M_{\Omega_{l,n}}y\right).$$

A validation loss value $L_{\mathrm{val}}^{\left(l\right)}$ is calculated every *l*th epoch to monitor the training process and determine the early stopping criteria of the training phase [20]:

(7) $$L_{\mathrm{val}}^{\left(l\right)}=L\left(M_{\Omega_{v}}\mathrm{FG}\left(M_{\Omega \setminus \Omega_{v}}y;\theta^{\left(l\right)}\right),M_{\Omega_{v}}y\right),$$

where $G\left(M_{\Omega \setminus \Omega_{v}}y;\theta^{\left(l\right)}\right)$ denotes the reconstructed output image after a full unrolled network forward pass using only the non‐validation samples $\Omega \setminus \Omega_{v}$ as input.

### 2.4 Time‐Conditioned ULF‐ZS‐SSL

Although the single‐element coil configuration in ULF MRI presents challenges for image reconstruction, the relatively small data size makes a full 3D approach computationally feasible. Moreover, the data acquisition uses two phase‐encoding directions (k_y_, k_z_), resulting in a 3D k‐space sampling trajectory with undersampling distributed across the k_y_‐k_z_ plane. Therefore, a 3D reconstruction approach can potentially stabilize the zero‐shot optimization by leveraging volumetric spatial correlations and significantly accelerate convergence compared to the original 2D approach [20]. Figure 1 illustrates the proposed physics‐informed unrolled network, ULF‐ZS‐SSL, which operates directly on in‐house 3D‐acquired ULF data.

Conventional unrolled network designs use shared convolutional weights across all unrolled iterations to limit model complexity, causing the network to apply the same static function at every step. In the proposed ULF‐ZS‐SSL, each unrolled iteration k is viewed as a discrete time step $t_{k}$. By conditioning the network on $t_{k}$, we introduce a non‐stationary mechanism that explicitly informs the shared parameters of their position within the unrolled optimization sequence. This enables the network to dynamically modulate its feature statistics across iterations, shifting its focus from coarse structure recovery to detail refinement [21]. This conditioning reduces the burden on the network to implicitly infer its optimization stage from the input alone, which can stabilize gradients and improve convergence efficiency. This is particularly impactful in the zero‐shot setting, where the network must learn reconstruction priors from a single image volume during inference‐time optimization. To encode iteration‐dependent information, we introduce a sinusoidal time‐step embedding similar to positional encodings used in transformer [22] and diffusion [23] models.

The embedding vector $e_{k}\in {\mathbb{R}}^{d}$ is defined using a set of exponentially‐spaced frequencies. Let d denote the embedding dimension with $d_{h}=d/2$ and $T_{\max}$ the maximum period. The frequencies are given by:

(8) $$\omega_{i}=\exp\left(-\frac{\ln\left(T_{\max}\right)}{d_{h}-1}i\right),\;i=\left\{0,\ldots ,d_{h}-1\right\}.$$

The time‐step embedding $e_{k}$ is then constructed by pairing sine and cosine functions:

(9) $$e_{k}=\left[\sin\left(\omega_{0}t_{k}\right),\ldots ,\sin\left(\omega_{d_{h}-1}t_{k}\right),\cos\left(\omega_{0}t_{k}\right),\ldots ,\cos\left(\omega_{d_{h}-1}t_{k}\right)\right].$$

For each unrolled iteration k, the embedding vector $e_{k}$ is passed through a fully‐connected layer to project it to the required channel dimension, allowing injection into the network via addition with the initial feature maps of each regularization block (Figure 1c).

### 2.5 ULF‐ZS‐SSL‐TL for Improved Reconstruction Speed

While ULF‐ZS‐SSL is completely training‐free, convergence can become slow for large 3D datasets. To address this limitation, we implement a TL extension [20] (ULF‐ZS‐SSL‐TL), where the network is first pretrained on a small set of fully‐sampled ULF datasets using the supervised objective in Equation (4) and then fine‐tuned on the target undersampled data in a zero‐shot manner using Equation (6).

## 3 Methods

### 3.1 Model Architecture, Hyperparameter Tuning, and Pretraining

The proposed ULF‐ZS‐SSL and ULF‐ZS‐SSL‐TL frameworks were implemented in PyTorch using a physics‐informed unrolled architecture composed of alternating learned regularization and DC steps (Figure 1b). Each unrolled iteration consisted of a 3D ResNet prior followed by a DC update solving Equation (3) via 10 conjugate‐gradient iterations. Complex‐valued data were represented as separate real and imaginary channels. The ResNet comprised an input convolution, multiple residual blocks with two 3 × 3 × 3 convolutions and LeakyReLU activation with a 0.1 slope, and an output convolution (Figure 1c). A residual scaling factor of 0.1 was applied to stabilize optimization. Sinusoidal time‐step embeddings (Section 2.4) were projected to the correct dimensionality, spatially broadcasted, and added to the initial feature maps of each regularization block to enable iteration‐dependent conditioning, thereby improving convergence speed and stability during the optimization. Given the single‐coil acquisition and undersampling across the k_y_‐k_z_ plane, reconstruction was performed in 3D as shown in Figure 1. A 2D slice‐wise alternative in the readout direction (k_x_) was also evaluated but resulted in substantially increased reconstruction time as shown in Figure S1.

To ensure a consistent and computationally efficient reconstruction across subjects, the number of unrolled iterations (*K*) and residual blocks (*B*) were empirically tuned prior to the experiments. The selected configuration of *K* = 5 and *B* = 5 provided the best balance between reconstruction accuracy, measured by SSIM and PSNR, and computational efficiency (Figure S2). Mixed‐precision training was performed for a maximum of 100 epochs using a normalized L1‐L2 loss [20], the Adam optimizer (learning‐rate = 0.0005, *β*_1_ = 0.9, *β*_2_ = 0.999), and batch‐size = 1 on a single NVIDIA L40S GPU (48 GB). Training was terminated early if the validation loss failed to improve for five consecutive epochs.

For the TL variant (ULF‐ZS‐SSL‐TL), pretrained weights were obtained from 22 fully‐sampled ULF brain datasets comprising IR‐T_1_w, T_1_w, and T_2_w contrasts (66 volumes total). Pretraining was conducted in a supervised manner (Equation 4) using retrospective undersampling of the fully‐sampled data to generate the training pairs, employing the same architecture as ULF‐ZS‐SSL. Separate pretraining runs were performed for acceleration factors *R* = 2 and *R* = 4, and the resulting weights were stored independently. Data augmentation was performed by using random affine transformations and random flips applied at each epoch. Pretraining was performed for a maximum of 100 epochs with early stopping and required approximately 20–30 min per acceleration factor.

### 3.2 Retrospective Undersampling Experiments

To assess reconstruction performance, retrospective undersampling was applied to fully‐sampled data at simulated acceleration factors *R* = 2, 3, 4, 5. These experiments enabled consistent comparison across all methods under controlled sampling conditions. Variable‐density Poisson‐disc sampling masks were generated for the phase‐encoding dimensions with a fully‐sampled central calibration region. Reconstruction accuracy was quantified using structural similarity (SSIM) and peak signal‐to‐noise ratio (PSNR), computed relative to fully‐sampled reference volumes. All metrics were evaluated consistently across the retrospective experiments for both brain and wrist datasets.

### 3.3 In‐Vivo Acquisition and True Undersampling Experiments

All in vivo measurements were performed on a custom‐built 47 mT Halbach‐based ULF MRI system (bandwidth = 20 kHz) using a single transmit/receive solenoid coil. Five healthy volunteers were scanned with informed consent obtained according to the rules of the local institutional ethics review boards.

#### 3.3.1 Brain Imaging

Three volunteers underwent brain imaging using a 3D TSE sequence with the following contrasts:

- IR‐T_1_w: TR/TE/TI = 1200/20/90 ms, echo‐train length 8.
- T_1_w: TR/TE = 600/16 ms, echo‐train length 8;
- T_2_w: TR/TE/TE_eff_ = 2500/20/150 ms, echo‐train length 15;

All scans were acquired with a spatial resolution of 1.5 × 1.5 × 5 mm^3^ and FOV of 205 × 225 × 190 mm^3^. Each volunteer was scanned at acceleration factors *R* = 1, 2, 4. The corresponding scan times for IR‐T_1_w/T_1_w/T_2_w acquisitions at *R* = 1 were 9:46/4:53/10:55 min, 5:30/2:48/6:07 min at *R* = 2, and 3:04/1:38/3:25 min at *R* = 4. The TI value of the IR‐T_1_w scan was chosen to maximize contrast between white and gray matter based on their T_1_ values at low‐field.

#### 3.3.2 Wrist Imaging

Two additional volunteers were scanned using a T_1_w 3D TSE protocol (TR/TE = 600/16 ms, echo‐train length 8). Fully‐sampled scans were performed in the coronal orientation with a spatial resolution of 1 × 1 × 3 mm^3^ and FOV of 150 × 180 × 90 mm^3^. For one volunteer, additional data were acquired with acceleration factors of *R* = 2 and *R* = 4. The corresponding scan times for the acquisitions with *R* = 1, 2, 4 were around 4:13/2:27/1:22 min.

## 4 Results

Per‐dataset optimization generally converged within 3–4 min with TL, compared with 8–10 min without it, depending on the acceleration factor and contrast. The time‐conditioned unrolling strategy provided stable training behavior across all experiments. Comparative results with and without time‐step embeddings are presented in Figure S3. Notably, the time‐step embeddings improved convergence speed by approximately 23% but had limited impact on the final reconstruction quality.

**FIGURE 2:**
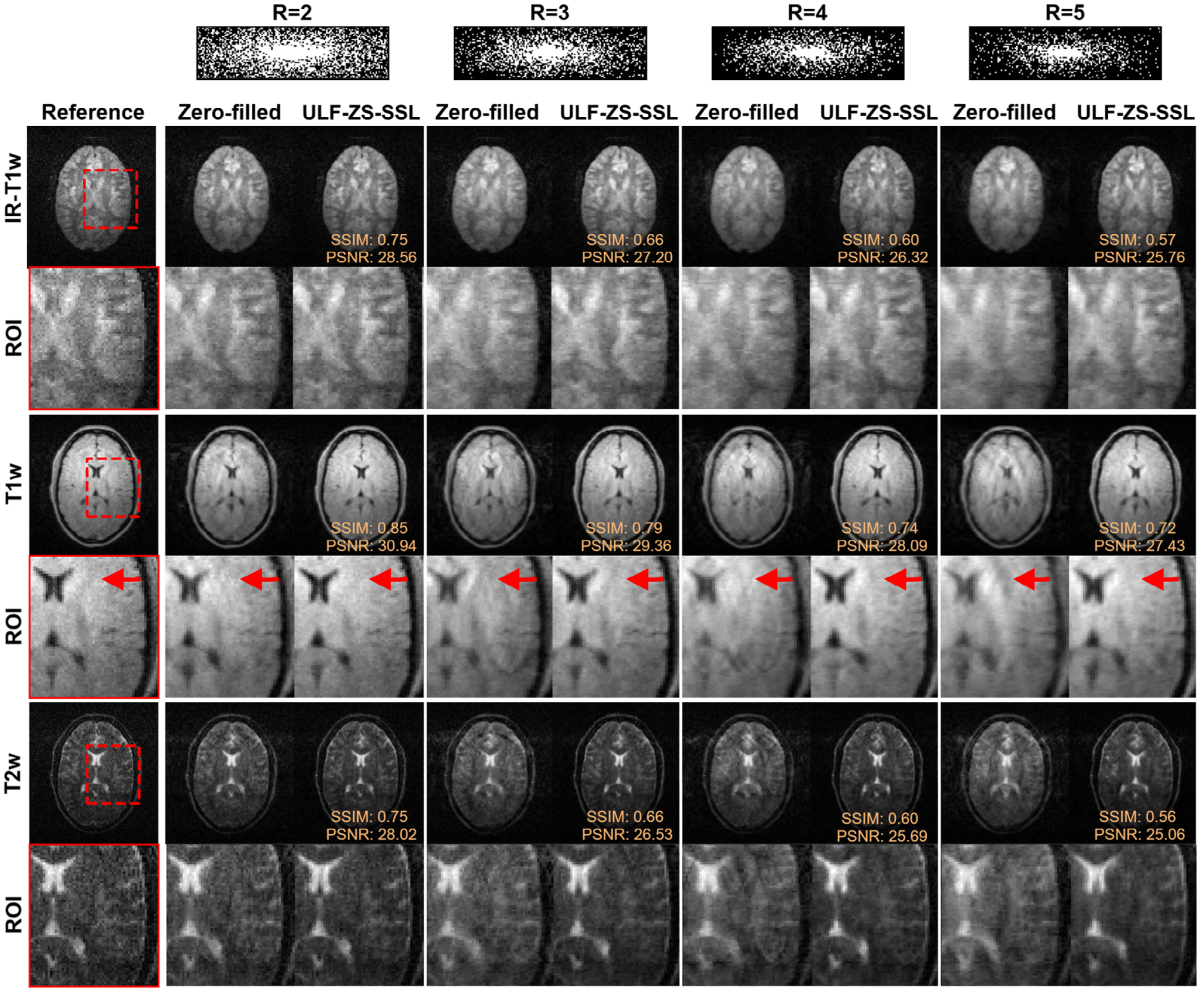
Comparison of the zero‐filled and ULF‐ZS‐SSL reconstructions across various retrospective acceleration factors (R). The top row displays the sampling masks for *R* = 2, 3, 4, 5. Subsequent rows present the fully‐sampled reference, the undersampled zero‐filled input, and the proposed ULF‐ZS‐SSL output for IR‐T_1_w, T_1_w, and T_2_w contrasts. Alternating rows show magnified regions of interest (ROIs) corresponding to the red dashed boxes. Quantitative metrics (SSIM and PSNR) are overlaid on the proposed reconstructions. Although the metrics decrease with higher acceleration factors, ULF‐ZS‐SSL still preserves strong perceptual fidelity and structural consistency. Red arrows indicate regions where undersampling artifacts are effectively suppressed.

As shown in Figure 2, for IR‐T_1_w, T_1_w, and T_2_w contrasts, ULF‐ZS‐SSL yielded consistent improvements over the zero‐filled inputs for accelerations *R* = 2, 3, 4, 5. Region‐of‐interest (ROI) zooms (red boxes) indicate better delineation of cortical ribbon/CSF boundaries and periventricular detail. Red arrows highlight visibly reduced undersampling artifacts relative to the inputs. Quantitative metrics (SSIM and PSNR) gradually decline as the acceleration factor increases since the optimization problem becomes more challenging.

**FIGURE 3:**
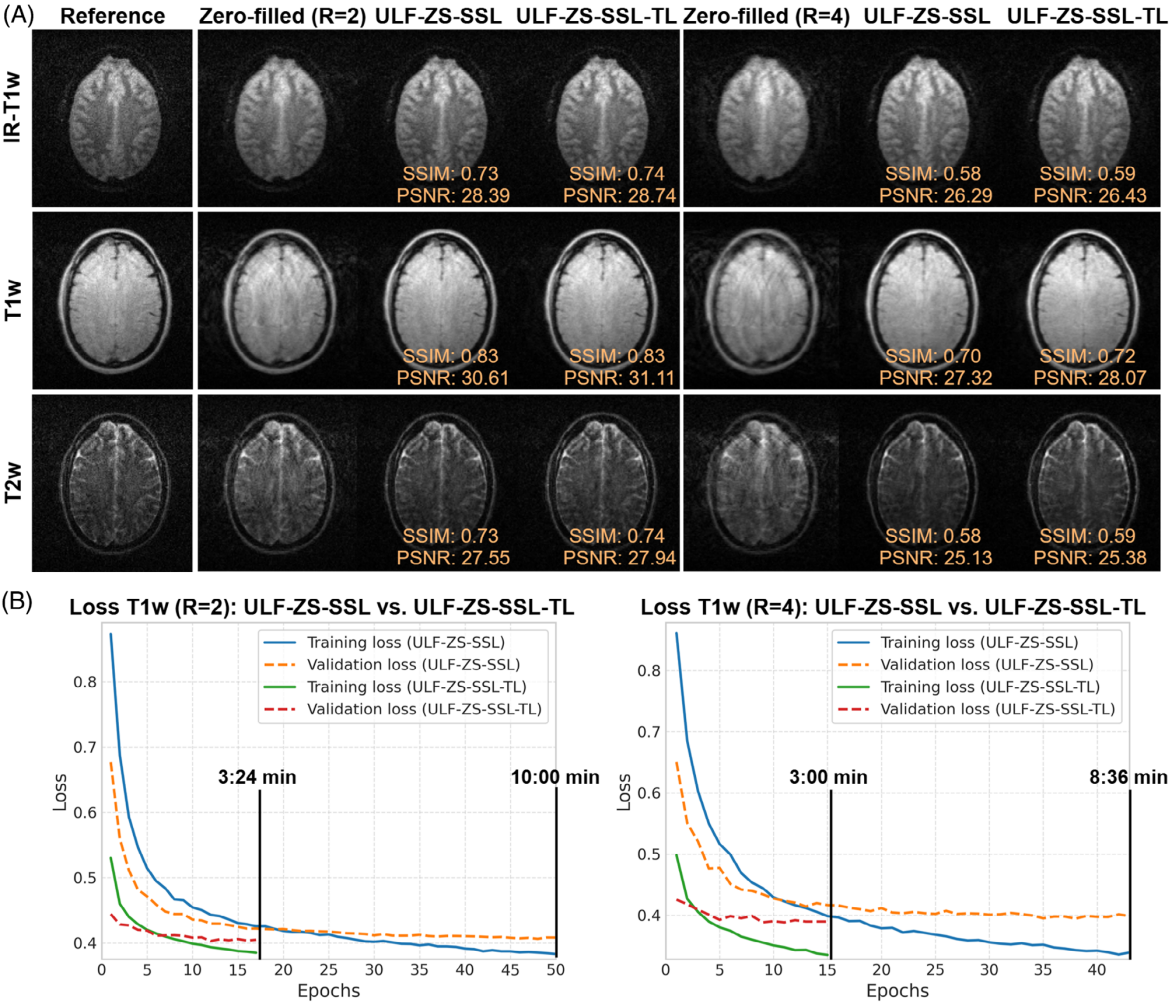
Assessment of reconstruction performance and convergence with and without transfer learning (TL). (a) Visual comparison of IR‐T_1_w, T_1_w, and T_2_w contrasts across acceleration factors *R* = 2 and *R* = 4. The ULF‐ZS‐SSL‐TL model, initialized with pretrained weights derived from a small dataset of 66 image volumes with data augmentation, produces image quality that is comparable to or slightly better than ULF‐ZS‐SSL. (b) Training and validation loss curves for T_1_w reconstructions at *R* = 2 (left) and *R* = 4 (right). The blue and orange curves represent standard weight initialization, while the green and red curves represent TL initialization. Vertical black lines mark the early‐stopping epoch and show that ULF‐ZS‐SSL‐TL converges more rapidly. The reconstruction time for a full 3D volume on an NVIDIA L40S GPU decreases from approximately 10 min to about 3 min.

ULF‐ZS‐SSL‐TL improved convergence, reducing reconstruction time by approximately a factor of three, while maintaining and, in some cases, slightly improving, SSIM and PSNR, as shown in Figure 3. For T_1_w reconstructions, training/validation loss curves show early‐stopping at 3.4 min versus 10.0 min for *R* = 2 and 3.0 min versus 8.6 min for *R* = 4 (with vs. without TL). Qualitatively, ULF‐ZS‐SSL‐TL preserves fine cortical detail and suppresses undersampling artifacts as effectively as ULF‐ZS‐SSL, indicating that the pretrained initialization provides a warm starting point for zero‐shot optimization while retaining subject‐specific adaptation.

**FIGURE 4:**
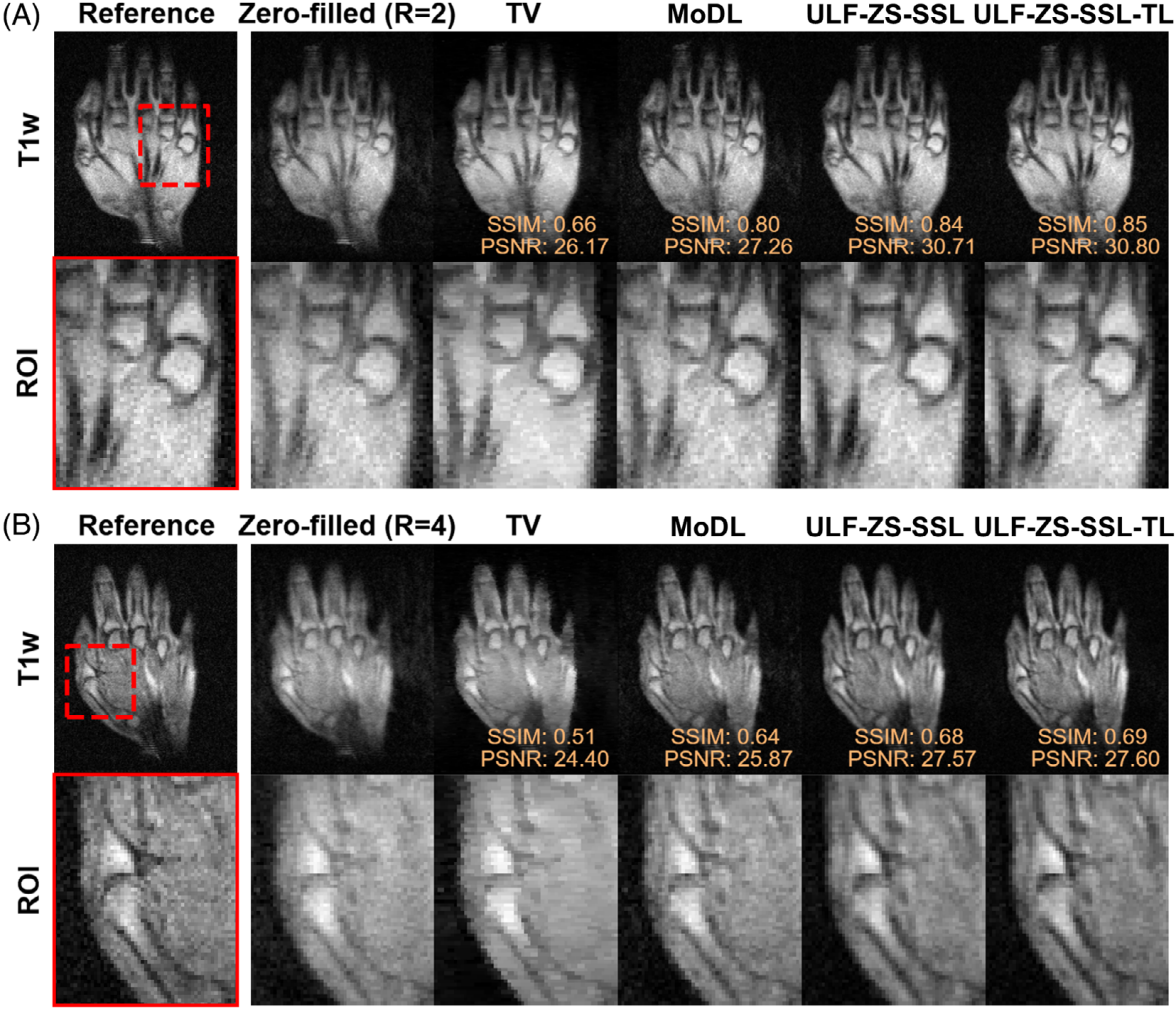
Evaluation of out‐of‐domain generalization using retrospectively undersampled T_1_w wrist scans from two subjects: (a) Subject 1 with *R* = 2 and (b) Subject 2 with *R* = 4. Despite being pretrained exclusively on brain data, ULF‐ZS‐SSL‐TL accelerates convergence and achieves reconstruction quality comparable to ULF‐ZS‐SSL. Both proposed methods outperform conventional TV and the supervised MoDL baselines, the latter likely limited by anatomical and contrast mismatches from the small brain dataset.

Figure 4 shows results of a retrospective undersampling experiment on T_1_w wrist scans (two subjects) to evaluate out‐of‐domain performance. Despite being pretrained on brain data, ULF‐ZS‐SSL‐TL accelerated convergence and improved reconstruction quality over (1) ULF‐ZS‐SSL (slightly), (2) TV, and (3) supervised MoDL trained only on brain data. The supervised MoDL baseline underperformed potentially due to anatomical or contrast mismatch, whereas ULF‐ZS‐SSL‐TL still leveraged the pretrained features as weight initialization for faster optimization and then adapted zero‐shot to wrist‐specific contrast and anatomy. True undersampled wrist reconstructions are shown in Figure S4.

**FIGURE 5:**
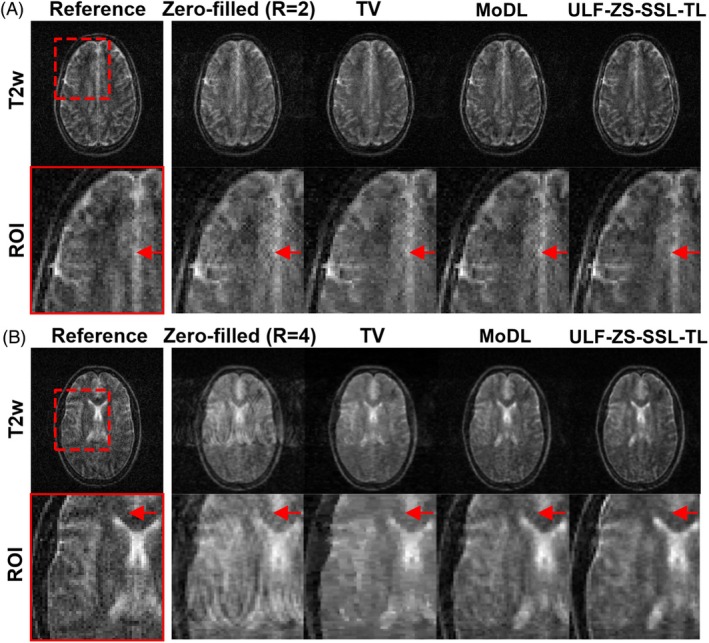
Comparison of reconstruction methods applied to true undersampled ULF brain data from two subjects with T_2_w contrast, which is typically the most challenging to reconstruct due to relatively lower SNR: (a) Subject 1 at *R* = 2 and (b) Subject 2 at *R* = 4. Columns display the fully‐sampled reference image, the zero‐filled input, and reconstructions using TV, supervised MoDL, and the proposed ULF‐ZS‐SSL‐TL model. Compared with the other reconstruction techniques, ULF‐ZS‐SSL‐TL recovers fine structural details more effectively (red arrows).

In the true undersampling experiment on the most challenging contrast (T_2_w) with the lowest SNR, as illustrated in Figure 5, ULF‐ZS‐SSL‐TL outperformed conventional TV and MoDL at both *R* = 2 and *R* = 4 (two subjects) in suppressing undersampling artifacts. The supervised MoDL, which is identical to the pretrained model used to initialize ULF‐ZS‐SSL‐TL, reduced some aliasing but still exhibited residual undersampling artifacts, suggesting sensitivity to domain mismatch and ULF noise characteristics. ULF‐ZS‐SSL‐TL more efficiently recovered brain structures with fewer residual artifacts.

## 5 Discussion

The proposed zero‐shot self‐supervised learning framework (ULF‐ZS‐SSL) demonstrates that high‐quality ULF MRI reconstruction can be achieved without any pre‐existing training data. In comparison, the supervised MoDL baseline [14], trained using the same architecture and identical data constraints to ensure fairness, shows lower performance likely due to the difficulty of learning a globally generalizable prior from a limited ULF dataset with strong subject‐to‐subject variability. By optimizing directly on the undersampled k‐space of a single subject in a zero‐shot manner, the network adapts to the unique signal, noise, and artifact characteristics of each scan, providing an advantage that is particularly beneficial in the low‐SNR ULF setting. Furthermore, transferring the network pretrained on brain datasets to wrist reconstruction also highlights the robustness of the framework to domain shifts, demonstrating that the network can leverage learned features for initialization while relying on zero‐shot adaptation to even handle unseen anatomies.

To ensure clinical viability, time‐conditioned unrolling efficiently incorporates iteration information with minimal computational overhead, improving convergence speed. When combined with the TL initialization, the model converges even faster, reducing per‐subject optimization to roughly 3 min and increasing clinical value. The cross‐anatomy evaluation on wrist data further confirms that pretraining on brain data can still accelerate convergence and enhance reconstruction quality in different anatomies when followed by subject‐specific zero‐shot refinement. Conceptually, one can view supervised, self‐supervised, and zero‐shot strategies as different pathways toward an optimal set of network parameters for a given reconstruction problem. In ULF‐ZS‐SSL‐TL, supervised pretraining can accelerate convergence toward this solution by providing a strong initialization, while the subsequent zero‐shot optimization refines the weights in a subject‐specific manner, potentially reaching a more accurate and data‐consistent reconstruction.

From a practical perspective, the proposed framework is well suited for portable or resource‐limited environments, as it enables subject‐specific zero‐shot reconstruction to be performed immediately after acquisition, eliminating the need for centralized data storage or retraining. The observed generalization of a modest pretrained model (with only 66 datasets) across anatomies (e.g., validation in wrist MRI) suggests that small, low‐field datasets combined with self‐adaptation may be sufficient for reliable deployment in diverse imaging settings. Although experiments were conducted on an NVIDIA L40S GPU for efficiency, the proposed framework does not inherently require high‐end hardware, and future benchmarking on more affordable consumer‐grade GPUs will be important to quantify performance‐cost trade‐offs.

Despite these encouraging results, several limitations remain. Firstly, this study did not compare performance with a broader range of state‐of‐art deep‐learning reconstruction frameworks, such as SSDU [19], transformer‐based approaches [22] and diffusion models [23]. A more general performance assessment would require a broader benchmarking study across architectures and undersampling patterns. However, for complicated architectures such as transformer and diffusion models, the amount of ULF datasets may not be enough to support reasonable training. Using simulation data adapted from high field (e.g., 3 T FastMRI data [24]) might be an option but is out of scope of this work and can be investigated in future research. Secondly, the study lacks radiologist evaluation. A reader study assessing perceived quality, diagnostic confidence, and feasible acceleration factors would help determine clinically acceptable trade‐offs between image quality and scan time. Third, architectural extensions, for example, incorporating attention mechanisms or transformer blocks, may theoretically enhance performance. However, the computational burden and GPU memory requirements could be challenging for 3D reconstruction. On the other hand, more complicated architectures may not be suitable for the zero‐shot learning concept, as they are not only computationally inefficient but also complicate the optimization landscape without yielding proportional gains in reconstruction quality for single‐subject tasks. This behavior is demonstrated in Figure S2, which shows that increasing the number of unrolls or residual blocks leads to a plateau in model performance. Finally, clinical validation on patient data remains essential, particularly to assess lesion detectability and the potential for false‐negatives, although physics‐based DC likely mitigates lesion loss.

It is also worth noting that recent studies have demonstrated the efficacy of denoising approaches [25, 26] for low‐field MRI. While the current work focuses on the inverse problem of reconstructing images from undersampled k‐space, integrating these advanced denoising techniques as a postprocessing stage could further enhance image quality. Investigating the synergy between physics‐informed reconstruction and subsequent deep learning‐based denoising remains a promising direction for future ULF research and needs further investigation.

## 6 Conclusion

In this work, we presented a time‐conditioned zero‐shot self‐supervised learning framework to accelerate 3D‐acquired ULF MRI (ULF‐ZS‐SSL). By combining physics‐based DC, sinusoidal time‐step embeddings, and optional TL initialization, the proposed method reconstructs high‐quality 3D images directly from undersampled single‐coil data without requiring external training datasets. The ULF‐ZS‐SSL‐TL variant with pretraining‐initialized network weights helps to achieve faster optimization convergence, enabling subject‐specific reconstructions within 3 min. The demonstrated performance on both brain and wrist data highlights the potential of this adaptive zero‐shot self‐supervised learning approach for robust and scalable reconstruction in portable and resource‐limited low‐field MRI systems.

## Conflicts of Interest

Peter Börnert is an employee of Philips.

## Supporting information


**Figure S1:** Comparison of 2D and 3D zero‐shot reconstruction for 3D‐acquired ULF MRI. (a) T1‐weighted reconstructions obtained from the same dataset are shown for a fully‐sampled reference, the zero‐filled model input (*R* = 2), 2D slice‐wise ULF‐ZS‐SSL, and 3D volumetric ULF‐ZS‐SSL. Quantitative metrics (SSIM, PSNR) indicate very similar reconstruction quality for both approaches. Despite using a batch size of 1, the 3D model converged substantially faster (8:24 min) than the 2D slice‐wise approach (23:48 min) with a batch size of 128. To enable slice‐wise ULF‐ZS‐SSL, the convolution operations in the network were changed from 3D to 2D. (b) Training and validation loss curves for the 2D and 3D models. The 3D approach shows a smoother and more stable convergence, whereas the 2D model plateaus in less epochs due to the larger batch dimension. Although 3D convolutions are computationally more intensive, the strong volumetric inductive bias stabilizes the optimization landscape, allowing the network to reach the target solution significantly faster, thereby accelerating the overall reconstruction time.
**Figure S2:** Hyperparameter sensitivity analysis for the ULF‐ZS‐SSL model. Heatmaps display SSIM (left) and PSNR (right) across varying numbers of unrolls and residual blocks, evaluated on retrospectively undersampled T1w data (*R* = 2). The highlighted configuration of 5 unrolls and 5 residual blocks (red box) was selected to optimize the trade‐off between quantitative performance and computational efficiency. Further increases in model capacity yielded diminishing returns for the zero‐shot self‐supervised setting.
**Figure S3:** Impact of sinusoidal time‐step conditioning in ULF‐ZS‐SSL. (a) Quantitative comparisons of reconstruction quality (SSIM and PSNR) between models trained with and without time‐step embeddings. Data are reported as mean ± standard deviation across 10 independent runs with different random seeds. No significant difference in final image quality is observed. (b) Training and validation loss curves (mean ± standard deviation) reveal faster convergence during initial epochs when time‐step embeddings are incorporated (black arrow). On average, this results in approximately 23% fewer iterations to reach the early stopping criterion when using time‐step embeddings (37 epochs, 7:24 min), compared to the baseline without embeddings (48 epochs, 9:36 min). Across the 10 runs, the maximum training time with time‐step conditioning was 8:24 min (42 epochs) compared to 12:00 min (60 epochs) without time‐step embeddings.
**Figure S4:** Reconstruction results of the proposed ULF‐ZS‐SSL‐TL framework for true undersampled ULF wrist data (T1w) from a single subject at acceleration factors *R* = 2 and R = 4. The results show a recovery of structural details compared to the fully sampled scan. Some anatomical details may look slightly different due to small motion between the scans.

## Data Availability

To support reproducible research, the source code used in this study, along with an ultra‐low‐field human brain dataset, has been made publicly available. The code and data can be found in the following repository: https://github.com/MartvStraten/ULF_3D_ZS_SSL.
