# Supplementary material for: Time‐Conditioned Zero‐Shot Self‐Supervised Reconstruction for Accelerated 3D Ultra‐Low‐Field MRI
Source: Magn Reson Med. 2026 Apr 28;96(3):1303–12. doi: 10.1002/mrm.70407 (PMC13327438; doi:10.1002/mrm.70407)
Supplement: Supplementary file 1 — Figure S1: Comparison of 2D and 3D zero‐shot reconstruction for 3D‐acquired ULF MRI. (a) T1‐weighted reconstructions obtained from the same dataset are shown for a fully‐sampled reference, the zero‐filled model input (R = 2), 2D slice‐wise ULF‐ZS‐SSL, and 3D volumetric ULF‐ZS‐SSL. Quantitative metrics (SSIM, PSNR) indicate very similar reconstruction quality for both approaches. Despite using a batch size of 1, the 3D model converged substantially faster (8:24 min) than the 2D slice‐wise approach (23:48 min) with a batch size of 128. To enable slice‐wise ULF‐ZS‐SSL, the convolution operations in the network were changed from 3D to 2D. (b) Training and validation loss curves for the 2D and 3D models. The 3D approach shows a smoother and more stable convergence, whereas the 2D model plateaus in less epochs due to the larger batch dimension. Although 3D convolutions are computationally more intensive, the strong volumetric inductive bias stabilizes the optimization landscape, allowing the network to reach the target solution significantly faster, thereby accelerating the overall reconstruction time. Figure S2: Hyperparameter sensitivity analysis for the ULF‐ZS‐SSL model. Heatmaps display SSIM (left) and PSNR (right) across varying numbers of unrolls and residual blocks, evaluated on retrospectively undersampled T1w data (R = 2). The highlighted configuration of 5 unrolls and 5 residual blocks (red box) was selected to optimize the trade‐off between quantitative performance and computational efficiency. Further increases in model capacity yielded diminishing returns for the zero‐shot self‐supervised setting. Figure S3: Impact of sinusoidal time‐step conditioning in ULF‐ZS‐SSL. (a) Quantitative comparisons of reconstruction quality (SSIM and PSNR) between models trained with and without time‐step embeddings. Data are reported as mean ± standard deviation across 10 independent runs with different random seeds. No significant difference in final image quality is observed. [file MRM-96-1303-s001.docx]

# Supporting Information


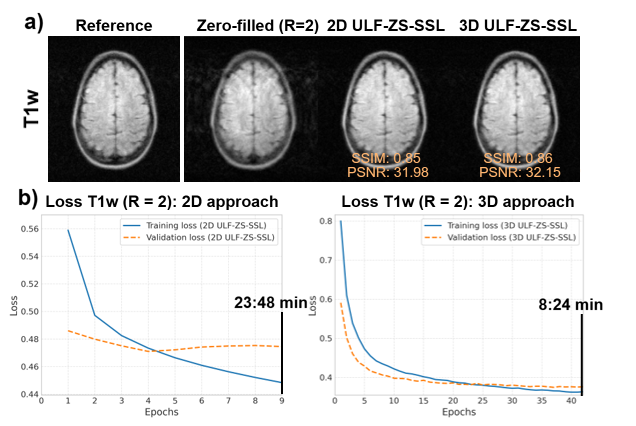


Figure S1: Comparison of 2D and 3D zero-shot reconstruction for 3D-acquired ULF MRI. a) T1-weighted reconstructions obtained from the same dataset are shown for a fully-sampled reference, the zero-filled model input (R=2), 2D slice-wise ULF-ZS-SSL, and 3D volumetric ULF-ZS-SSL. Quantitative metrics (SSIM, PSNR) indicate very similar reconstruction quality for both approaches. Despite using a batch size of 1, the 3D model converged substantially faster (8:24 minutes) than the 2D slice-wise approach (23:48 minutes) with a batch size of 128. To enable slice-wise ULF-ZS-SSL, the convolution operations in the network were changed from 3D to 2D. b) Training and validation loss curves for the 2D and 3D models. The 3D approach shows a smoother and more stable convergence, whereas the 2D model plateaus in less epochs due to the larger batch dimension. Although 3D convolutions are computationally more intensive, the strong volumetric inductive bias stabilizes the optimization landscape, allowing the network to reach the target solution significantly faster, thereby accelerating the overall reconstruction time.


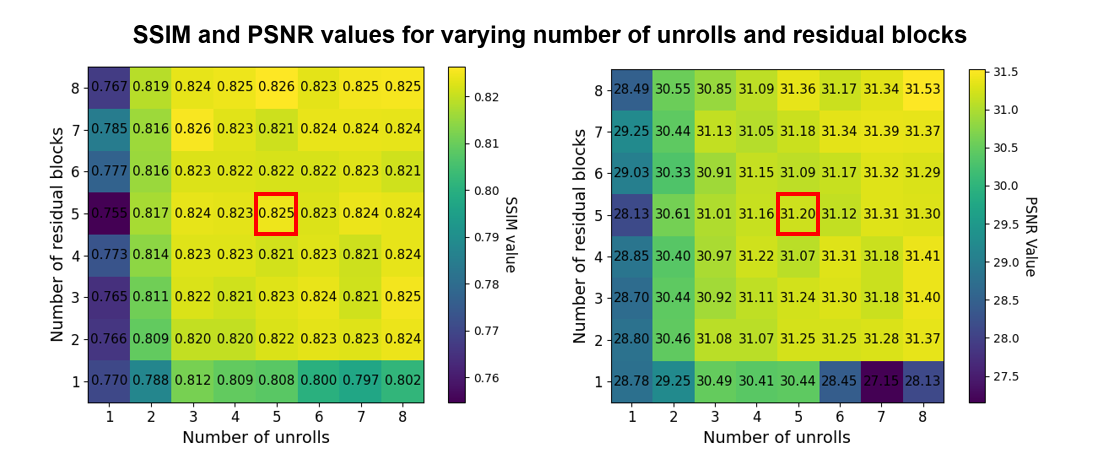


Figure S2: Hyperparameter sensitivity analysis for the ULF-ZS-SSL model. Heatmaps display SSIM (left) and PSNR (right) across varying numbers of unrolls and residual blocks, evaluated on retrospectively undersampled T1w data (R=2). The highlighted configuration of 5 unrolls and 5 residual blocks (red box) was selected to optimize the trade-off between quantitative performance and computational efficiency. Further increases in model capacity yielded diminishing returns for the zero-shot self-supervised setting*.*


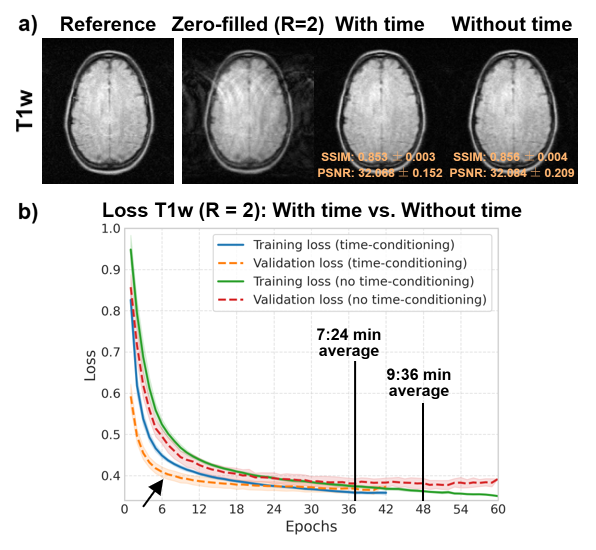


Figure S3: Impact of sinusoidal time-step conditioning in ULF-ZS-SSL. a) Quantitative comparisons of reconstruction quality (SSIM and PSNR) between models trained with and without time-step embeddings. Data are reported as mean ± standard deviation across 10 independent runs with different random seeds. No significant difference in final image quality is observed. b) Training and validation loss curves (mean ± standard deviation) reveal faster convergence during initial epochs when time-step embeddings are incorporated (black arrow). On average, this results in approximately 23% fewer iterations to reach the early stopping criterion when using time-step embeddings (37 epochs, 7:24 minutes), compared to the baseline without embeddings (48 epochs, 9:36 minutes). Across the 10 runs, the maximum training time with time-step conditioning was 8:24 minutes (42 epochs) compared to 12:00 minutes (60 epochs) without time-step embeddings.


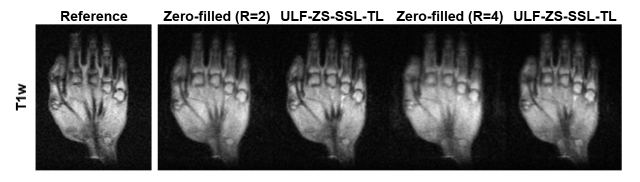


Figure S4: Reconstruction results of the proposed ULF-ZS-SSL-TL framework for true undersampled ULF wrist data (T1w) from a single subject at acceleration factors R=2 and R=4. The results show a recovery of structural details compared to the fully sampled scan. Some anatomical details may look slightly different due to small motion between the scans*.*
